# Insulin Induces Outer Blood–Retinal Barrier Disruption via Downregulation of Claudin-19

**DOI:** 10.1167/iovs.67.4.39

**Published:** 2026-04-17

**Authors:** Karen R. Hernandez, Lana M. Pollock, Aidan D. Rodriguez, M. F. Lohr, Ryota L. Matsuoka, Bela Anand-Apte

**Affiliations:** 1 Department of Pharmacology, Case Western Reserve University, Cleveland, Ohio, United States; 2 Department of Ophthalmic Research, Cole Eye Institute, Cleveland Clinic Foundation, Cleveland, Ohio, United States; 3 Department of Neurosciences, Cleveland Clinic Foundation, Cleveland, Ohio, United States

**Keywords:** blood–retinal barrier, diabetic retinopathy, retinal pigment epithelium

## Abstract

**Purpose:**

Diabetic retinopathy is a common complication of diabetes mellitus, a disease that is reaching epidemic proportions worldwide. Although diabetic macular edema has generally been attributed to breakdown of the inner blood–retinal barrier, accumulating evidence suggests that the outer blood–retinal barrier (oBRB) may also be involved. Clinical studies have shown that acute intensive insulin therapy causes a transient worsening of diabetic retinopathy in type 1 and type 2 diabetes. In this study, we tested the hypothesis that insulin directly disrupts the oBRB by targeting claudin-19 tight junctions in the retinal pigment epithelium (RPE).

**Methods:**

The effects of insulin on claudin-19 tight junctions in primary RPE cells were assessed by immunohistochemistry and western blot analysis using in vitro cell culture models and an in vivo transgenic zebrafish model. Changes in blood–retinal barrier integrity were quantified using electric cell-substrate impedance sensing (ECIS).

**Results:**

Claudin-19 was identified as the predominant claudin in primary porcine RPE cells and was essential for maintaining oBRB integrity. Barrier function did not differ between RPE cells cultured under physiological (5 mM) or high (25 mM; diabetic) glucose conditions. In contrast, insulin treatment disrupted the oBRB independently of glucose concentration. Insulin significantly reduced claudin-19 protein levels without affecting transcript abundance, indicating post-transcriptional regulation. Consistent with the in vitro findings, insulin induced claudin-19 tight junction disruption in vivo in a transgenic zebrafish model expressing claudin-19 fused to enhanced green fluorescent protein. Consistent with the in vitro findings, high glucose alone did not disrupt claudin-19 in vivo.

**Conclusions:**

These findings demonstrate that insulin disrupts the oBRB independently of glucose concentration in both in vitro and in vivo models. This work provides new insight into the molecular mechanisms underlying early worsening of diabetic retinopathy and highlights a potential role for hyperinsulinemia in type 2 diabetes–associated retinal pathology.

Diabetic macular edema (DME) is the leading cause of vision loss in diabetes, caused by fluid accumulation in the retina due to blood–retinal barrier (BRB) disruption. Although much attention has focused on inner BRB disruption in diabetes, growing evidence from humans and animal models suggests that retinal pigment epithelium (RPE) alterations play a critical role in DME pathogenesis.^1^^–^^5^ The RPE is a monolayer of pigmented epithelial cells that separates the neuronal retina from the choriocapillaris. Its apical membrane faces photoreceptor outer segments, and its basolateral membrane faces Bruch's membrane, collectively forming the outer blood–retinal barrier (oBRB). The RPE regulates the exchange of nutrients, waste products, and metabolic signals between the retina and systemic circulation. Gap junctions, adherens, and tight junctions on the apical surface restrict paracellular diffusion, with paracellular resistance being about tenfold higher than transcellular resistance, thus classifying the RPE as a tight epithelium. Glucose transporters located on both the apical and basolateral membranes supply glucose to photoreceptors, and excess water produced by the high metabolic turnover of the retina is removed via transcellular transport through aquaporin-1. The RPE phagocytoses photoreceptor outer segments containing photodamaged proteins, radicals, and lipids, a process tightly coordinated with photoreceptors. Therefore, RPE health is essential for preserving the homeostatic environment required for normal retinal physiology and visual function.^6^^,^^7^

Claudins are tetraspanin transmembrane proteins with 27 established members to date. A combination of specific claudins is expressed in unique tissues/species, and these claudins form the backbone of tight junctions by modulating the barrier or channel properties.^8^ Claudin-19 is the predominant claudin in human RPE cells and is also highly expressed in the kidney. Mutations in *CLDN19* and *CLDN16* cause the renal disease familial hypomagnesemia with hypercalciuria and nephrocalcinosis (FHHN) but patients with mutations in *CLDN19* also develop ocular involvement (FHHNOI), a syndrome that includes bilateral macular coloboma and chorioretinal degeneration.^9^

Insulin use has recently been reported to be a significant risk factor for diabetic retinopathy,^10^^–^^12^ observed between 3 months and 3 years after starting insulin treatment.^13^ Although often attributed to insulin-induced transient hypoglycemia, the underlying pathophysiology remains unclear. We investigated the role of claudin-19 in maintaining the integrity of tight junctions in the RPE under normal and diabetic conditions. Given the critical function of claudin-19 in RPE barrier formation, we hypothesized that diabetic stressors, such as elevated glucose and insulin levels, disrupt claudin-19 expression and localization, compromising the RPE barrier. Specifically, we examined the effects of high glucose and high insulin on the structural and functional integrity of RPE tight junctions, with a focus on alterations in claudin-19.

## Materials and Methods

### Isolation of Primary Porcine Retinal Pigment Epithelial Cells

Eyes were obtained from a local abattoir (3D Meats, Dalton, OH, USA) and processed within 3 to 4 hours postmortem using a modified protocol.^14^ Extraocular tissue was trimmed, and globes were placed in cold PBS with 1000-U/mL penicillin–streptomycin and then incubated in 0.2% iodine (APL82277K; Medline, Northfield, IL, USA) for 10 minutes. Eyes were rinsed, transferred to a sterile hood, and placed in cold PBS with 1000-U/mL penicillin–streptomycin. The anterior segment and vitreous were removed. Eyecups were incubated in 1-mM PBS/EDTA at 37°C and 5% CO_2_ for 30 minutes. Retinas were removed, and eyecups were incubated in Gibco Trypsin-EDTA (0.25%), phenol red (25200114; Fisher Scientific, Waltham, MA, USA) for 90 minutes under the same conditions. RPE cells were dislodged by pipetting and transferred to 15-mL tubes with Dulbecco's Modified Eagle Medium (DMEM) (prepared by the institutional media core) plus 10% Gibco Fetal Bovine Serum (FBS; A5256801; Fisher Scientific), centrifuged (1200 rpm) for 5 minutes, and resuspended in DMEM, 10% FBS, 50-U/mL penicillin–streptomycin, and 0.001% ciprofloxacin (61314-656-05; Sandoz, Basel, Switzerland). Cells were pooled, filtered through a 100-µm strainer, and counted using a hemocytometer.

### Quantitative Real-Time Reverse-Transcription PCR

RPE cells were collected at harvest or seeded in 12-well plates (6.0 × 10^6^ cells/well) and cultured in DMEM (institutional media core) supplemented with 10% Gibco FBS, 0.001% ciprofloxacin, and 50-U/mL penicillin–streptomycin (institutional media core). Upon reaching confluence, cells were maintained in 1% FBS for an additional week. Total RNA was extracted using the RNeasy Midi Kit (74104; QIAGEN, Hilden, Germany) and reverse-transcribed with Invitrogen SuperScript VILO Master Mix (11755250; Fisher Scientific). Quantitative real-time reverse-transcription PCR (RT-qPCR) was performed using SYBR Green (QS1005; Alkali Scientific, Fort Lauderdale, FL, USA), with glyceraldehyde 3-phosphate dehydrogenase (GAPDH) as the normalization control. Relative mRNA expression was calculated using the 2^−^^Δ^^ΔCt^ method. The following primers were used:

Claudin-1 forward: GCAGCTTCTTGCTTCTCAACT
Claudin-1 reverse: CCATAGCTCTGGCTCAAGGG
Claudin-3 forward: CTACGACCGCAAGGACTACG
Claudin-3 reverse: TAGCATCTGGGTGGACTGGT
Claudin-5 forward: GGCGACTACGACAAGAAGAACT
Claudin-5 reverse: CAAAGTCTGGTCTGGGCACA
Claudin-11 forward: CTTGCCGAGCCCTGATGAT
Claudin-11 reverse: ACAGAGAGCCAGCAGAATGAG
Claudin-19 forward: GTGGCTGGGTGGGTATCATC
Claudin-19 reverse: GAGCTGACTGGATGTGACCTT
GAPDH forward: AGGTCGGAGTGAACGGATTTG
GAPDH reverse: CGTGGGTGGAATCATACTGGAA

### Immunostaining of Porcine RPE Wholemount

Tissue was fixed in 100% methanol (20 minutes at –20°C), washed with PBS, permeabilized with 0.3% Triton X-100 (20 minutes at room temperature), and blocked in 2% BSA/PBS for 1 hour. Samples were incubated overnight at 4°C with claudin-19 (NBP1-59277, 1:500; Novus Biologicals, Centennial, CO, USA) and ZO-1 Monoclonal Antibody (ZO1-1A12) (33-9100, 1:500; Thermo Fisher Scientific, Waltham, MA, USA). Tissue was washed with PBS, incubated with Invitrogen secondary antibodies (A-11008, 1:1000; A-11005, 1:1000; Thermo Fisher Scientific) for 1 hour at room temperature. After washes, 4′,6-diamidino-2-phenylindole (DAPI; D9542; 1:1000 in PBS; Sigma-Aldrich, St. Louis, MO, USA) was applied for 10 minutes. Tissue was mounted in glass-bottom dishes (150680; Thermo Fisher Scientific) with VECTASHIELD (VectorLabs, Newark, CA, USA) and 1% low-melt agarose. *Z*-stack images were acquired on a Leica TCS SP8 confocal microscope (Leica Microsystems, Wetzlar, Germany) using a 63× oil objective.

### Western Blot

Cells were lysed in RIPA Lysis and Extraction Buffer (89901; Thermo Fisher Scientific) with Halt Protease Inhibitor Cocktail (78429; Thermo Fisher Scientific), sonicated, and centrifuged. Supernatant was analyzed using a Pierce BCA Protein Assay Kit (23227; Thermo Fisher Scientific). Then, 10 µg of protein was used for sodium dodecyl sulfate–polyacrylamide gel electrophoresis (SDS-PAGE), and proteins were transferred overnight to a polyvinylidene fluoride (PVDF) membrane. Membranes were blocked for 1 hour (927-80001; LICORbio, Lincoln, NE, USA) at room temperature and then incubated with primary antibody claudin-19 (NBP1-59277, 1:500; Novus Biologicals) and β-actin (3700s, 1:5000; Cell Signaling Technology, Danvers, MA, USA) overnight at 4°C. They were then incubated with secondary antibody (IRDye, 1:10,000; LICORbio) for 1 hour at room temperature and imaged using the Odyssey cLX Imager (LICORbio).

### Electric Cell-Substrate Impedance Sensing Impedance Testing

Electric cell-substrate impedance sensing (ECIS) 8W10E+ PET arrays (Applied Biophysics, Troy, NY, USA) were pretreated with 10-mM l-cysteine (15 minutes at room temperature) and washed twice with Milli-Q water (MilliporeSigma, Burlington, MA, USA). After the addition of complete media, a baseline impedance reading was taken, and 1.5 × 10^5^ cells were seeded per well. When resistance had stabilized, treatment (small interfering RNA [siRNA] knockdown or glucose insulin) was initiated.

### siRNA Experiments

Transient knockdown of claudin-19 was achieved using siRNA (Integrated DNA Technologies, Coralville, IA, USA): forward, rCrArGrGrUrGrCrArGrUrGrCrArArGrCrUrCrUrArCrGrACT; reverse, rArGrUrCrGrUrArGrArGrCrUrUrGrCrArCrUrGrCrArCrCrUrGrArC (positions 206–231). A non-targeting siRNA (DharmaFECT, D-0011810-10-20; Horizon Discovery, Cambridge, UK) was used as control. Cells were incubated in serum-free media for 2 hours. Then, 25-nM siRNA was diluted in 100 µL serum and antibiotic-free DMEM and incubated for 5 minutes. Separately, DharmaFECT 4 (T-2004-02; Horizon Discovery) was diluted in 100 µL of the same medium (0.5% v/v) for 5 minutes. The siRNA and diluted DharmaFECT were combined and incubated for 20 minutes at room temperature, and 200 µL of transfection mixture was added to each well.

### Insulin Treatment

Primary porcine RPE cells were seeded (6.0 × 10^5^ cells/well) onto 12-well plates and cultured in 1× DMEM 25-mM glucose (11-500; LRI Media Core) supplemented with 10% Gibco FBS, 50 U/mL penicillin–streptomycin (721-500, 5000 U/mL; LRI Media Core), and 0.001% ciprofloxacin. They were then placed in a 37°C incubator with 5% CO_2_. At confluence, they were switched to 1% FBS before treatment with 5-mM or 25-mM glucose ± 100-nM human insulin (Novolin R, NDC 0169-1833-11, 100 units/mL; Novo Nordisk, Bagsværd, Denmark) for 72 hours with daily media changes.

### Junction Mapper Analysis

Six images (three per condition) were analyzed from two independent experiments comparing high glucose with and without 100-nM insulin totaling 1099 junctions. Images were processed using Junction Mapper software.^15^ The threshold for high-glucose samples was determined using the Otsu method in ImageJ 2.16.0/1.54p (National Institutes of Health, Bethesda, MD, USA).

### Generation of Tg(rpe65a:cldn19-EGFP) Zebrafish

A transgenic zebrafish line, Tg(rpe65a:cldn19-EGFP), was generated using the Tol2kit system. The *claudin19* gene was PCR-amplified from wild-type zebrafish genomic DNA (sense: CTCAGTGTTGTCGGCAGAA; antisense: ATGAGACGGCTGTCAAAGTG). The PCR product was separated on a 1% agarose gel, excised, and purified using the QIAquick Gel Extraction Kit (28704; QIAGEN). The PCR product was cloned into the pDONR221 vector to generate middle entry clones containing *attP2R/P3* sites. Recombination reactions (10 µL) were transformed into Invitrogen One Shot TOP10 Chemically Competent *E. coli* (C404003; Thermo Fisher Scientific), heat shocked at 42°C for 30 seconds, immediately transferred to ice, and recovered in 250 µL super optimal broth with catabolite repression (SOC) medium at 37°C for 1 hour (200 rpm). Then, 100 µL was plated onto prewarmed Luria-Bertani (LB) agar plates with kanamycin. Colonies were selected, grown overnight in LB agar plates with kanamycin, and purified using the QIAprep Spin Miniprep Kit (27104; QIAGEN). The plasmid was verified by DpnI digestion and sequencing. For the final construct, 20 fmol each of P5E-rpe65a, p3a-EGFP_PA, pDestTol2CG2, and the pDONR221-clnd19 were combined using Invitrogen LR Clonase II Plus enzyme (Thermo Fisher Scientific). Constructs were transformed, validated, and sequenced as described above. Injection mix (1 nL) containing 500 ng plasmid and 250-ng/µL Tol2 transposase RNA was injected into one-cell stage embryos. Transgenic carriers were identified by enhanced green fluorescent protein (EGFP) expression in the heart via fluorescent stereomicroscopy.

### Zebrafish Husbandry

Zebrafish husbandry was performed under standard conditions in accordance with institutional and national ethical and animal welfare guidelines. All zebrafish work was approved by the Cleveland Clinic's Institutional Animal Care and Use Committee (protocol no. 00003285) and conformed to the ARVO Statement for the Use of Animals in Ophthalmic and Vision Research. Adult fish were maintained on a standard 14-hour light/10-hour dark daily cycle. Fish embryos/larvae were raised at 28.5°C. To prevent skin pigmentation, 0.003% 1-phenyl-2-thiourea (PTU; P5272; Sigma-Aldrich) was used.

### Live Zebrafish Imaging

For tile scans, fish were mounted laterally in 1% low-melt agarose gel on a glass-bottomed plate. Images were taken using a Leica TCS SP8 confocal laser scanning microscope with a 10× objective lens (dry) and a 0.75 zoom factor. Images were captured and processed using the Leica Application Suite X (LAS-X) software (version 3.7.0.20979).

### Wholemount Immunostaining

Zebrafish embryos were treated with 0.003% PTU. At 4 to 7 days post-fertilization (dpf), larvae were incubated in fish water containing 80-mM glucose, 0.8 U/mL insulin, a combination of glucose and insulin, or regular fish water. At 7 dpf, larvae were killed and fixed overnight in 4% PFA at 4°C. Eyes were dissected, treated with proteinase K (10 µg/mL for 15 minutes at room temperature), rinsed, and blocked in 0.2% BSA (2 hours at room temperature). Eyes were incubated with anti-GFP antibody (TP401, 1:1000; Torrey Pines Biolabs, Secaucus, NJ, USA) overnight at 4°C followed by incubation in Invitrogen secondary antibody (A-21207, 1:500; Thermo Fisher Scientific) overnight at 4°C. After washing, eyes were mounted in 1% low-melt agarose on glass-bottom dishes. *Z*-stack images were acquired on a Leica TCS SP8 confocal microscope using a 40× water objective (zoom factor 0.8).

### Zebrafish Tight Junction Quantification

Tight junction intensity per area (TJIA) measurements were performed using a series of ImageJ macros. Preprocessing consisted of white top-hat morphological filtering and a Gaussian blur filter, and the same threshold was applied to each image. Only the portion of the image that contained tight junction staining was used to measure the area and intensity.

### Zebrafish Western Blot

Twelve larvae per group were added to a clean 1.5-mL microcentrifuge tube with RIPA buffer and protease inhibitor. Samples were sonicated and centrifuged for 10 minutes at 14,000 rpm at 4°C. Then, 10 µg of protein was used for SDS-PAGE, and proteins were transferred overnight to a PVDF membrane. Membranes were blocked in 5% non-fat milk for 1 hour (1706404; Bio-Rad Laboratories, Hercules, CA, USA) at room temperature and then incubated with primary antibody claudin-19 (NBP2-92415, 1:500; Novus Biologicals) and β-actin overnight at 4°C. They were then incubated with IRDye secondary antibody for 1 hour at room temperature and imaged using the LICORbio Odyssey cLX Imager.

### Zebrafish Insulin ELISA

Insulin uptake was measured using an insulin ELISA (90095; Crystal Chem, Elk Grove Village, IL, USA). Six larvae per group were rinsed with PBS (three times), sonicated in 100-µL diluent buffer, and centrifuged. ELISA was performed per the manufacturer's instructions, and absorbance was read at A450 with A630 subtracted.

### Zebrafish Glucose Assay

Glucose uptake was measured using the Invitrogen Amplex Red Glucose/Glucose Oxidase Assay Kit (A22189; Thermo Fisher Scientific) following the manufacturer's instructions. Eight larvae per group were rinsed with PBS (three times), sonicated in 100-µL 1× reagent buffer, and then centrifuged. Supernatants were diluted 1:10. Absorbance was measured at 560 nm.

### Statistical Analysis

All experiments were repeated at least three times, and the results are represented as the mean ± SD. Statistical analyses were performed using Prism 10.4.2 (GraphPad, Boston, MA, USA) by two-tailed unpaired Student's *t*-test, one-way ANOVA, or two-way ANOVA.

## Results

### Claudin-19 Is the Predominant Claudin in Primary Porcine RPE Cells

**Figure 1.**
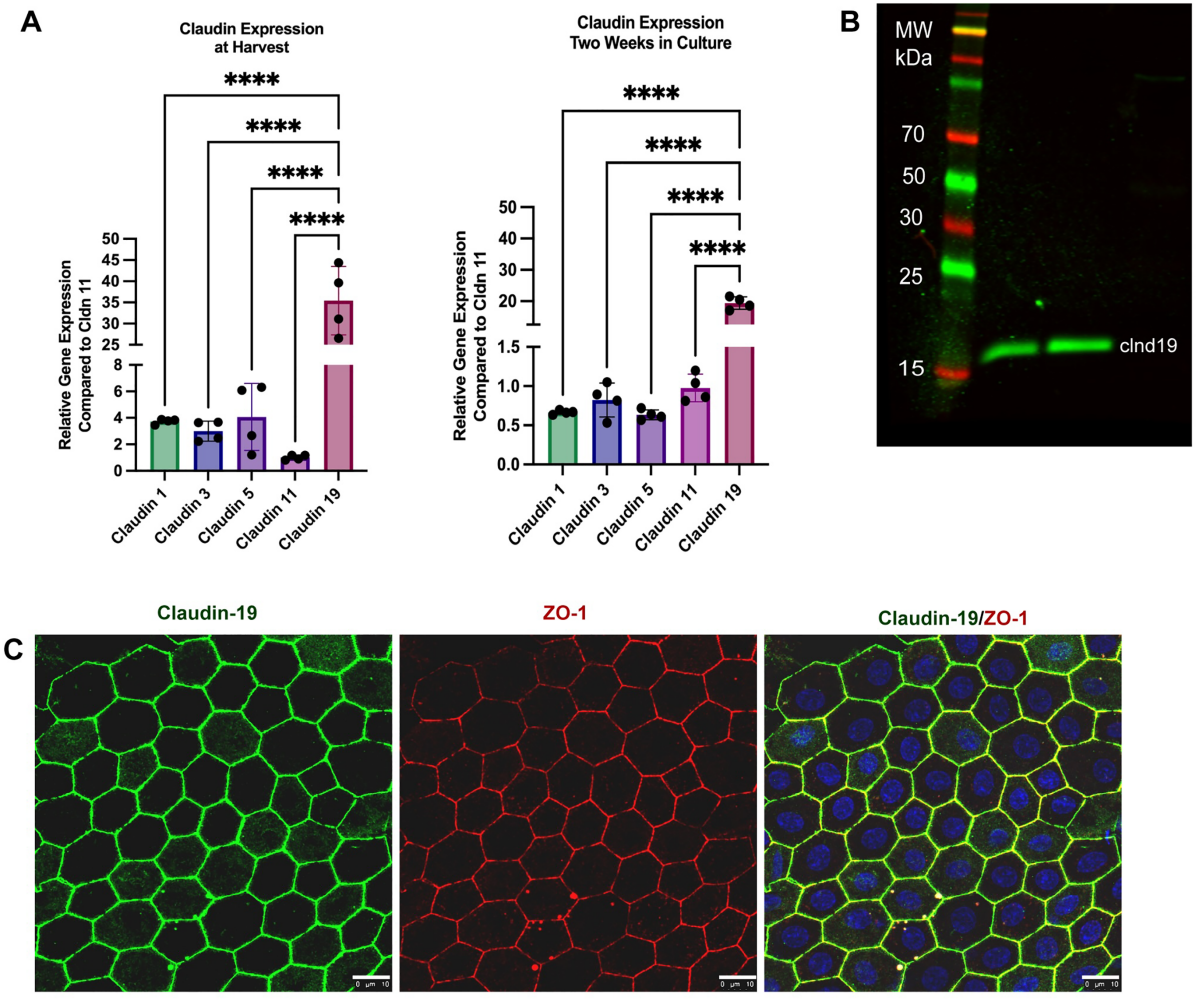
Claudin-19 is the claudin predominantly expressed in porcine RPE. (**A**) qPCR measuring claudin expression from cells collected at harvest and cells that were in culture for 2 weeks. *Bars* represent mean ± SD (*n* = 2). **P < 0.01; ****P < 0.0001 (one-way ANOVA with Brown–Forsythe test). (**B**) Western blot analysis of claudin-19 expression in primary porcine RPE cells that were in culture for 2 weeks (*n* = 2). (**C**) Immunofluorescent staining of claudin-19 (*green*) and ZO-1 (*red*) on porcine RPE tissue. *Scale bar*: 10 µm.

**Figure 2.**
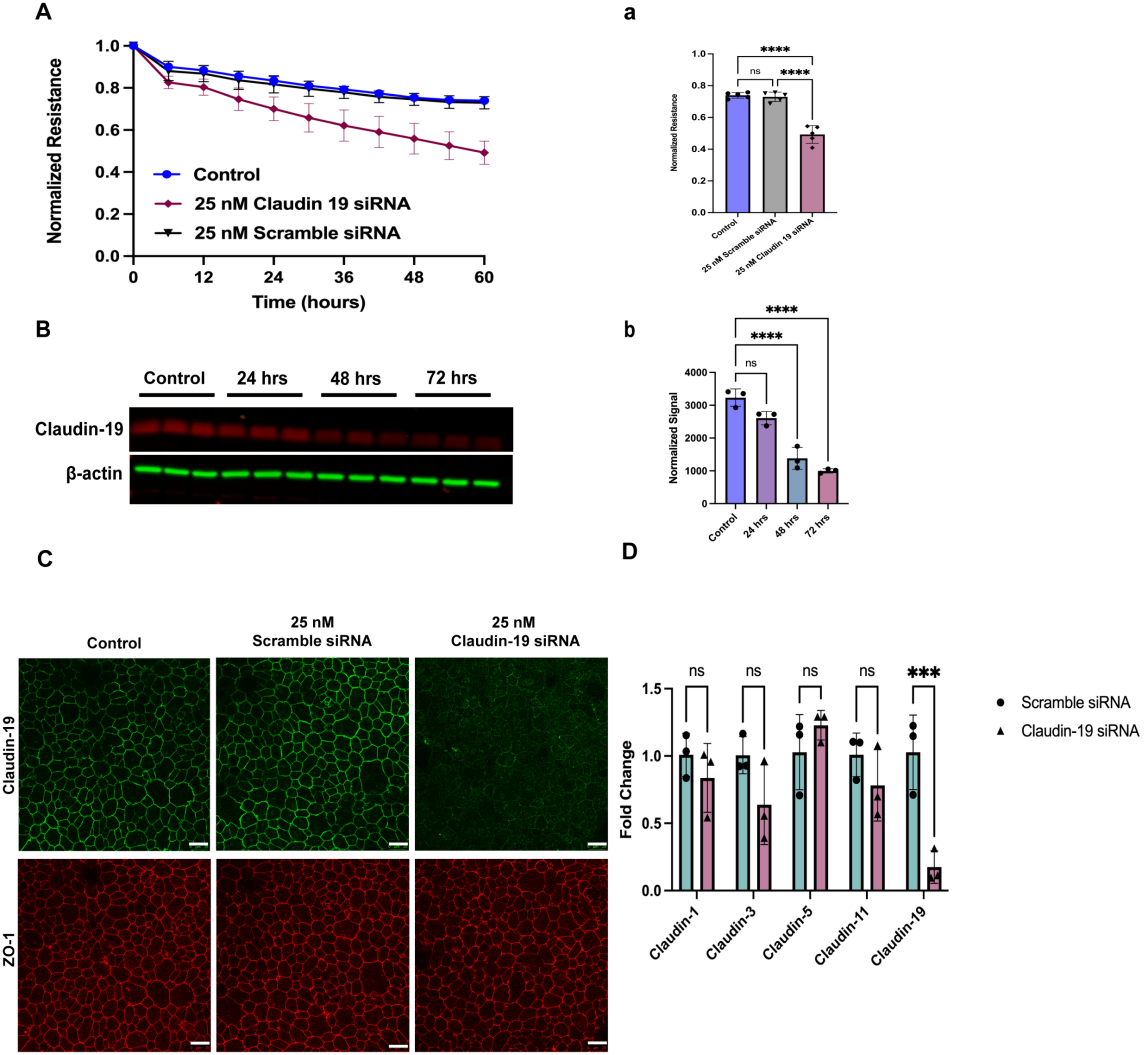
Claudin-19 plays an essential role in maintaining the outer BRB. (**A**) Real-time barrier-resistant measurement (2000 Hz) of primary porcine RPE cells treated with 25-nM scrambled siRNA or claudin-19 siRNA or were left untransfected. Only final points were quantified. Data are mean ± SD (*n* = 5). ****P < 0.0001 (ordinary one-way ANOVA with Tukey's test). (**B**) Western blot analysis of porcine RPE treated for 24, 48, and 72 hours with 25-nM scrambled siRNA or claudin-19 siRNA. β-Actin was used as a loading control. Data are mean ± SD (*n* = 5). ****P < 0.0001 (ordinary one-way ANOVA with Tukey's test). (**C**) Immunofluorescence staining of claudin-19 (*green*) and ZO-1 (*red*) in porcine RPE cells. *Scale bar*: 25 µm. (**D**) mRNA expression of claudin-1, claudin-3, claudin-5, claudin-11, and claudin-19 after 72-hour treatment with 25-nM scrambled siRNA or claudin-19 siRNA. Data are mean ± SD (*n* = 3). ***P < 0.0001 (two-way ANOVA with Tukey's test); ns, not significant.

To assess claudin expression in the RPE, RT-qPCR was performed on primary porcine RPE cells. RNA was extracted from cells either at harvest or after 2 weeks in culture. We examined epithelial barrier–associated claudins 1, 3, 5, 11, and 19. mRNA levels were normalized to GAPDH and compared to claudin-11, the least expressed. Claudin-19 mRNA was ∼30-fold higher than the others at harvest. Similarly, following 2 weeks in culture, claudin-19 expression remained elevated (Fig. 1A). We confirmed claudin-19 protein expression by western blot analysis and determined that, in porcine RPE, claudin-19 appears to display a molecular weight of 17 kDa (Fig. 1B). Immunofluorescence of wholemount porcine RPE tissue indicated that claudin-19 is localized at tight junctions, as revealed by zonula occludens‐1 (ZO-1) co-labeling (Fig. 1C). The antibody specificity was confirmed through knockdown experiments where we observed substantially reduced claudin-19 protein expression levels after treating RPE cells with siRNA, as shown in Figure 2.

### Claudin-19 Is Essential for RPE Barrier Function

To evaluate the role of claudin-19 in RPE barrier function, we used ECIS to monitor real-time barrier resistance after claudin-19 knockdown. When resistance had stabilized, RPE cells were transfected with 25-nM claudin-19 siRNA or scrambled control. Within 24 hours, a decline in resistance was observed in cells in which claudin-19 was downregulated, and by 60 hours there was a significant disruption in barrier function. The final readings at 60 hours post-transfection were plotted to evaluate barrier function (Fig. 2A). These results are consistent with previously reported data.^16^ The effective knockdown of claudin-19 was confirmed by western blot analysis (Fig. 2B), as well as immunofluorescent staining (Fig. 2C). Interestingly, ZO-1 remained intact (Fig. 2C). To test if other claudins compensated for claudin-19 loss, we performed RT-qPCR for claudins 1, 3, 5, and 11 in claudin-19 siRNA–treated cells and found no change in their mRNA levels (Fig. 2D). These results suggest that claudin-19 is the predominant claudin in the RPE and regulates barrier function

### Insulin Induces RPE Barrier (oBRB) Disruption

**Figure 3.**
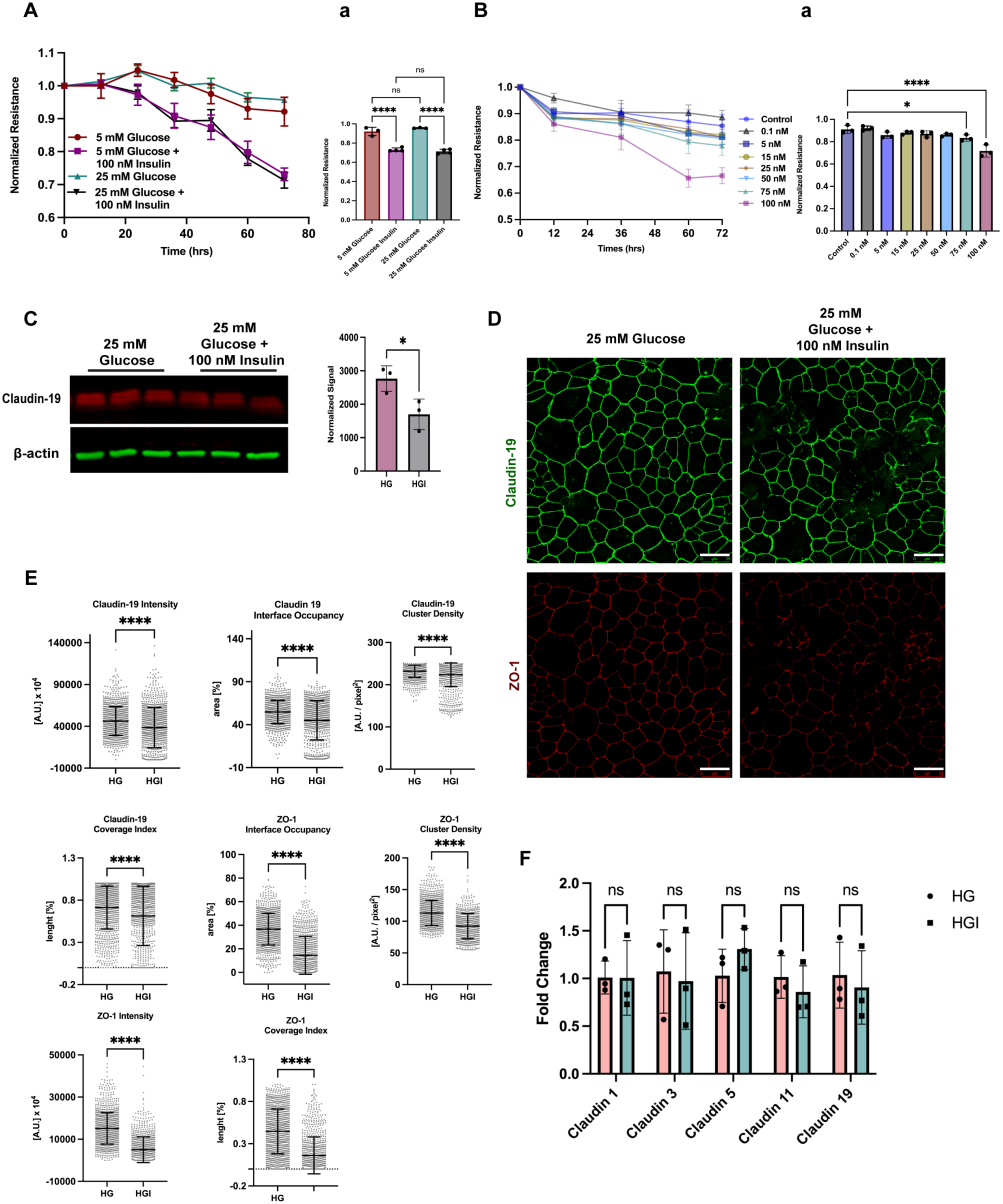
Insulin induces oBRB breakdown regardless of glucose concentration. (**A**) Real-time impedance of RPE monolayers treated with 5- or 25-mM glucose ± 100-nM insulin. Endpoint quantification is shown as mean ± SD (*n* = 3). ****P < 0.0001 (one-way ANOVA with Brown–Forsythe test and Tukey's test). (**B**) Dose-dependent effect of insulin (0.1–100 nM) on RPE impedance. Quantified endpoints are shown as mean ± SD (*n* = 3 or 4). *P < 0.05; ****P < 0.0001 (one-way ANOVA with Brown-Forsythe correction and Dunnett's test). (**C**) Western blot of RPE cells treated with 25-mM glucose ± 100-nM insulin, expressed as mean ± SD (*n* = 3). *P < 0.05 (unpaired two-tailed *t*-test). (**D**) Immunofluorescence for claudin-19 and ZO-1 under the same conditions. *Scale bar*: 25 µm. (**E**) Quantification of junctional parameters using Junction Mapper, expressed as mean ± SD (*n* = 1099). ****P < 0.0001 (unpaired *t*-test). (**F**) RT-qPCR of claudin transcripts normalized to GAPDH and inter-claudin expression, reported as mean ± SD (*n* = 3). (two-way ANOVA with Sidak's correction); ns, not significant. HG, high glucose; HGI, high glucose insulin.

To assess the direct effects of insulin on the oBRB, RPE cells were seeded on ECIS chips and cultured in 5-mM (normal) or 25-mM (high) glucose to mimic physiological or diabetic conditions. To control osmolarity, 20-mM l-glucose was added to 5-mM d-glucose medium. Interestingly, high glucose alone did not induce a decrease in resistance (Fig. 3A). However, exposure of RPE cells to 100-nM insulin resulted in a reduction in resistance under both normoglycemic and hyperglycemic conditions (Fig. 3A). An insulin dose–response assay determined that 75-nM and 100-nM doses of insulin could initiate barrier disruption (Fig. 3B). Insulin significantly reduced claudin-19 expression in RPE cells after 72-hour exposure, as shown by western blot (Fig. 3C). Immunofluorescence confirmed disrupted claudin-19 staining in insulin-treated RPE cells, whereas ZO-1 remained intact (Fig. 3D). Notably, insulin treatment induced cell swelling, which may result from increased intracellular Na^+^ and K^+^ caused by activation of Na^+^/H^+^ exchanges, Na^+^/K^+^/Cl^−^ cotransporters, and Na^+^/K^+^-ATPase activity.^17^ Junction Mapper,^15^ a semi-automated pipeline, was used to identify length, area, and intensity of junction markers. Insulin treatment confirmed the decrease in tight junction protein intensity in insulin-treated samples. The coverage index measures the percentage of the cell boundary that is covered by the junction marker staining. Insulin-treated cells showed a decrease for both claudin-19 and ZO-1 staining along the interface. These results indicate that insulin causes claudin-19 and ZO-1 disruption in RPE cells (Fig. 3E). To evaluate whether insulin impacts claudin-19 at the protein or transcript level, we performed RT-qPCR on RPE cells treated with insulin. No change in claudin-19 mRNA levels was detected between control and insulin-treated samples, indicating that insulin likely affects claudin-19 post-transcriptionally (Fig. 3F).

### Insulin Induces RPE Claudin-19 Disruption In Vivo

**Figure 4.**
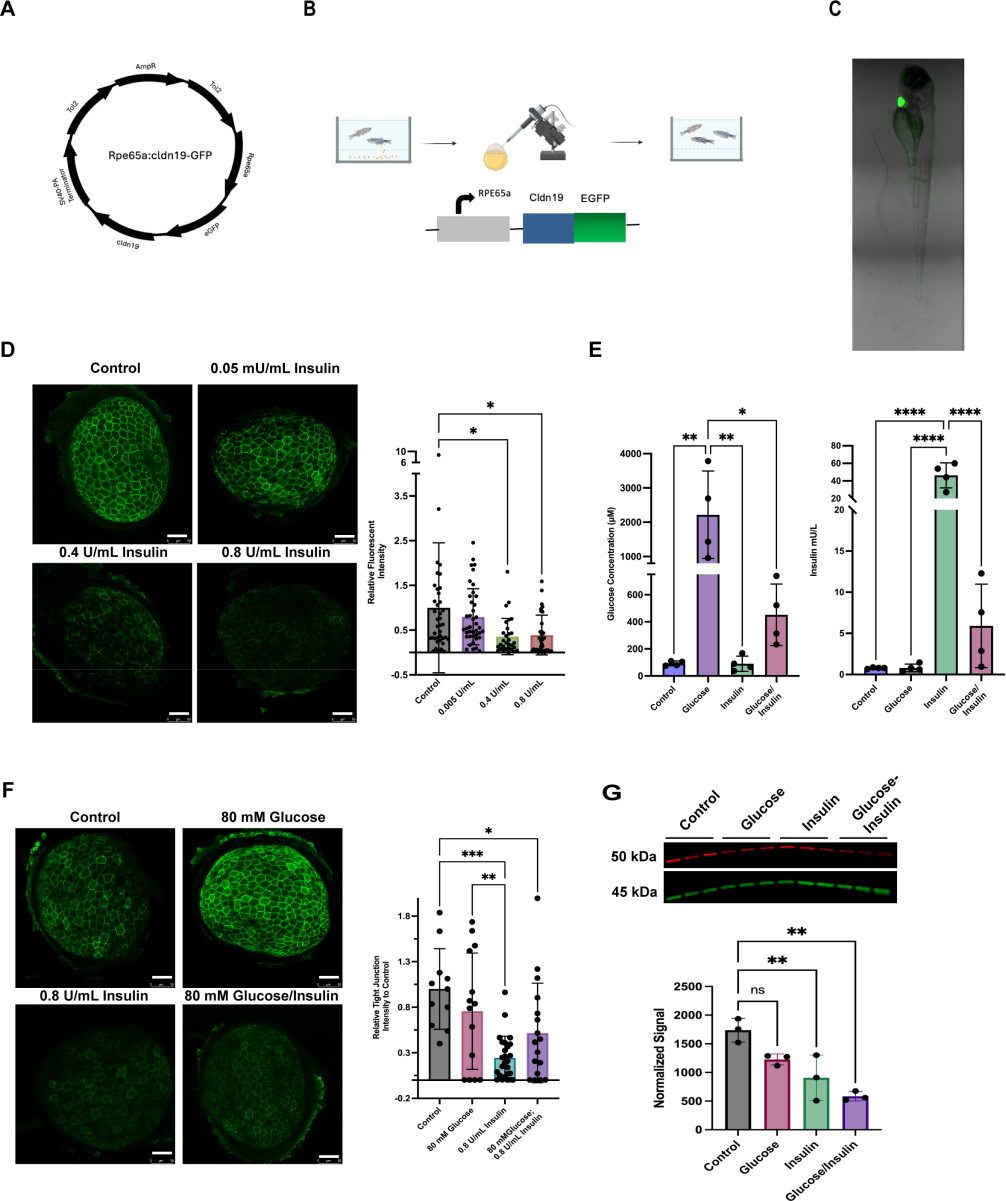
Insulin causes claudin-19 breakdown in vivo. (**A**) Schematic of the EGFP–claudin-19 construct. (**B**) Generation of Tg(rpe65a:cldn19-EGFP) transgenic zebrafish. (**C**) Transgenic larvae at 122 hours post-fertilization showed the EGFP heart marker indicating transgenesis. (**D**) Immunofluorescence of claudin-19 expression in the RPE after 72 hours under different insulin conditions. Quantification is shown at the *right*, with data expressed as mean ± SD (*n* = 31–40 eyes). *Scale bar*: 50 µm. *P < 0.0051 (one-way ANOVA with Barlett's test). (**E**) Glucose uptake (glucose assay) and insulin levels (ELISA), shown as mean ± SD (*n* = 4). *Scale bar*: 50 µm. Glucose uptake: *P < 0.0015; **P < 0.0015 (one-way ANOVA with Brown–Forsythe test). Insulin levels: ****P < 0.0001 (ELISA). (**F**) Claudin-19 expression in larvae under control, glucose, insulin, and insulin plus glucose. Each *dot* represents one eye. *Scale bar*: 50 µm. *P < 0.0051; **P < 0.00150; ***P < 0.0004 (ordinary one-way ANOVA with Brown–Forsythe test). (**G**) Western blot analysis of 6 dpf larvae under control, glucose, insulin, and insulin plus glucose treatment. *P < 0.03; **P < 0.01 (one way ANOVA with Tukey's multiple comparison test).

The oBRB is challenging to study in vivo due to its anatomical position in the eye and the lack of real-time, non-invasive imaging tools, resulting in a critical need for a reliable in vivo model. To address this unmet need, we generated a transgenic zebrafish line that expresses claudin-19 fused to an EGFP under the control of the RPE65a promoter (Fig. 4A). The Tol2 gene expression vector containing this transgene cassette was injected into one-cell stage embryos along with Tol2 transposase mRNA (Fig. 4B). Injected F0 fish were grown into adulthood and subjected to founder screening by outcrossing them with wild-type animals carrying no fluorescent reporter. The resulting F1 larvae were screened for the EGFP heart marker incorporated into the transgenic expression vector. We identified a founder whose progeny exhibited strong heart marker expression. Immunostaining of dissected eyes from these heart marker–positive larvae showed RPE-specific EGFP expression co-localized with the ZO-1 tight junction marker. These results validated EGFP–claudin-19 transgene localization at tight junctions in RPE cells, establishing a novel transgenic tool for visualizing claudin-19 tight junction integrity in live zebrafish (Fig. 4C). We have previously reported that zebrafish develop an intact BRB by 3 dpf.^18^ Based on this developmental timing of retinal barrier formation, we performed insulin dose–response experiments starting at 4 dpf, using the newly generated EGFP–claudin-19 transgenic line. Specifically, we treated the transgenic larvae from 4 to 7 dpf for 72 hours with different concentrations of insulin applied to the fish water to test whether they can induce outer BRB disruption. We observed strong RPE-specific EGFP–claudin-19 expression in control and 0.005-U/mL insulin-treated samples, whereas both 0.4-U/mL and 0.8-U/mL insulin treatments resulted in significantly reduced transgene expression (Fig. 4D). These results indicate that insulin induces dose-dependent changes in EGFP–claudin-19 expression in vivo, consistent with the results obtained from porcine RPE cell culture experiments (Fig. 3).

Next, we investigated how hyperglycemia and hyperinsulinemia conditions affect RPE-specific EGFP–claudin-19 expression and barrier integrity. A common method to induce hyperglycemia and hyperinsulinemia in zebrafish is to immerse the fish in a high-glucose and/or high-insulin solution.^19^ Thus, we treated larvae between 4 and 7 dpf under the following conditions: fish water (control), 80-mM glucose (hyperglycemia), 0.8-U/mL insulin (hyperinsulinemia), and a combination of 80-mM glucose and 0.8-U/mL insulin (hyperglycemia/hyperinsulinemia).

To validate the expected induction of hyperglycemia and hyperinsulinemia conditions, we measured glucose and insulin uptake levels using ELISA and the Invitrogen Amplex Red Glucose/Glucose Oxidase Assay. Larvae treated with insulin or the glucose/insulin combination solution showed higher insulin levels compared to control or glucose treatment alone. Larvae treated with 80-mM glucose also had increased glucose levels when compared to control or insulin treatment alone (Fig. 4E). Insulin, either alone or in combination with glucose, led to significant decreased EGFP–claudin-19 expression (Fig. 4F). Western blot analysis also showed a decrease in EGFP–claudin-19 fusion protein (Fig. 4G). Consistent with our in vitro data in porcine RPE cells, these results indicate that, at high concentrations, insulin reduces claudin-19 protein and may lead to barrier disruption. These experiments indicate that the insulin-induced turnover of claudin-19 occurs in an experimental animal model and is conserved between mammals and teleost fish.

## Discussion

DME characterized by accumulation of fluid in the macular region of the retina is a consequence of breakdown of the BRB. The prevailing belief is that hyperglycemia is the sole cause of all pathology; however, recent studies suggest that other factors may also play a crucial role. Although epidemiological studies and randomized clinical trials have indicated that glycemic control is crucial for preventing vascular complications of diabetes, insulin therapies aimed at controlling glucose metabolism do not prevent long-term complications.^20^^–^^22^ The Action to Control Cardiovascular Risk in Diabetes (ACCORD) trial revealed a statistically significant increase in overall mortality in the insulin-treated intensive glycemic control group, leading to the early termination of this arm of the study.^23^ Concurrently, the ACCORD eye study group reported that, although intensive glycemic therapy significantly reduced the risk of diabetic retinopathy progression, it did not lower the risk of moderate vision loss.^24^ In addition, DME appears to be more resistant to laser photocoagulation and anti–vascular endothelial growth factor (VEGF) therapies, suggesting that other factors may contribute to this complication. Therefore, understanding the mechanisms underlying DME is essential for developing effective therapeutic strategies.

Increased levels of exogenous insulin have been associated with a higher occurrence of DME in individuals with type 2 diabetes.^25^ The Epidemiology of Diabetes Interventions and Complications (EDIC) study found that patients undergoing intensive insulin therapy faced a greater risk of developing complications of DR, even when their glycosylated hemoglobin (HbA1C) levels were within the “normal” range.^26^^,^^27^ Long-term data revealed that the 10-year incidence of DME in patients with type 2 diabetes who were on insulin was 25%, compared to 14% in those not using insulin.^28^ This increase in DME among insulin users was observed regardless of the duration or severity of the disease, contrary to initial assumptions. Switching from oral medications to insulin in patients with non–insulin-dependent (type 2) diabetes mellitus has been linked to a significantly higher risk of retinopathy progression and visual impairment.^12^^,^^29^^,^^30^ Compounding this issue, some clinical trials have reported that initiating acute intensive insulin therapy in patients with long-standing poor glycemic control can lead to a temporary worsening of diabetic retinopathy.^31^^–^^37^ This phenomenon is believed to be associated with levels of insulin-like growth factor (IGF) and VEGF.^29^^,^^38^^–^^41^ Although it has been suggested that hypoglycemia as a consequence of intensive insulin therapy might induce inner BRB breakdown,^42^ previous reports have demonstrated that high-insulin/high-glucose treatment (as a model of hyperglycemia and hyperinsulinemia) synergistically reduces tight junction integrity in endothelial cells.^43^ Endothelial-specific insulin receptor substrate-1 (IRS-1) overexpression disrupts and worsens neurovascular integrity after hypoxic–ischemic injury via tight junction protein disassembly.^44^ These results suggest that insulin can have a direct effect on endothelial cell tight junctions.

Although studies evaluating the molecular mechanisms of BRB breakdown have focused mostly on the disruption of the vascular endothelial tight junctions, there is increasing evidence that the RPE barrier is likely to play a critical role, but it has been challenging to study. Leakage of albumin and fluorescein from the RPE near the macula has been observed in patients with diabetic retinopathy, even in the absence of cystic retinal changes, indicating RPE barrier disruption.^4^ Optical coherence tomography (OCT) studies have revealed that about one-third of DME cases exhibit serous detachment, which signifies fluid accumulation likely due to compromised RPE barrier function. Histological examinations of postmortem eyes from both diabetic and non-diabetic individuals have shown albumin leakage into the retina from both the inner and outer BRBs.^3^^,^^45^ In addition, animal models of diabetes demonstrate early loss of RPE barrier function, molecular evidence of tight junction protein damage, and VEGF and advanced glycation end product (AGE)-mediated ultrastructural damage and leakage of the RPE barrier.^46^^,^^5^ Our understanding of the molecular and cellular mechanisms involved in the pathogenesis of diabetic retinopathy and DME in both type 1 and type 2 diabetes is limited.

We tested the novel hypothesis that insulin disrupts claudin-19, leading to oBRB breakdown. Primary porcine RPE cells were used to examine the role of claudin-19 in oBRB function and its response to high glucose and insulin. Porcine eyes closely resemble human eyes in both structure and function, making them an ideal model for studying various retinal diseases. We identified claudin-19 as the predominant claudin at cell–cell tight junctions and showed that it co-localized with ZO-1. Interestingly, claudin-19 appears as a single band in Figure 1B, whereas double bands are observed in later western blots. This difference may reflect sample source, as Figure 1B used lysates collected from harvest, but subsequent blots used lysates from cultured cells. Double bands have been reported for other claudins and are commonly attributed to post-translational modifications.^47^ siRNA-mediated knockdown of claudin-19 reduced transepithelial resistance, indicating barrier disruption and tight junction disruption as seen by immunostaining, whereas ZO-1 remained unaffected. In addition, claudin-19 knockdown did not affect the expression of the other claudins, which were at insufficient levels to compensate. Our findings align with those of Liu et al.,^16^ who studied RPE cells differentiated from human induced pluripotent stem cells. We found no difference in barrier function in cells cultured in normal or high glucose, which suggests that hyperglycemia likely does not directly alter the oBRB. High insulin induced barrier breakdown regardless of glucose concentration, and no changes in claudin-19 mRNA expression suggest post-transcriptional regulation at the protein level. Although the relationship between claudin-19 and insulin remains unclear, prior studies have shown that insulin can induce post-translational modifications of tight junction proteins. In lymphatic endothelial cells, Gonzalez-Nieves et al.^48^ demonstrated that insulin treatment enriched palmitoylation of barrier integrity proteins such as claudin-5, small GTPases, and ubiquitination enzymes. Furthermore, insulin treatment promotes linear claudin-5 staining with increased trans-endothelial electrical resistance.^43^ In contrast, our results showed a downregulation of claudin-19 on RPE cells, highlighting cell-type specific effects of insulin and the need for further study of insulin–claudin-19 interactions in this context. Expression of claudins has previously been demonstrated to be regulated by growth factors such as epidermal growth factor (EGF) and transforming growth factor-β (TGF-β) by mechanisms unique to each claudin even in the same cell type.^49^^–^^51^ Because claudin-19 siRNA reduced TER without affecting ZO-1 morphology, but insulin reduced both claudin-19 and ZO-1, the role of ZO-1 in this process must be further evaluated. Future experiments with insulin receptor knockdown and/or identification of claudin-19 binding partners will strengthen the link among claudin-19, ZO-1, and insulin signaling.

Our in vivo studies also showed that insulin, alone or with glucose, could disrupt claudin-19 in zebrafish larvae, consistent with the in vitro results. Animal models of diabetes demonstrate early loss of RPE barrier function, molecular evidence of tight junction protein damage, and VEGF and advanced glycation end product (AGE)-mediated ultrastructural damage and leakage of the RPE barrier.^46^^,^^5^ We have demonstrated that insulin can have a direct effect on the outer blood–retinal barrier by disrupting claudin-19. Our findings, along with other studies in the literature, raise important questions about the role of insulin in regulating oBRB integrity. Additionally, this sets the stage for understanding the molecular mechanisms behind the “early worsening” observed with insulin therapy and/or DME in type 2 diabetes, where patients often experience hyperinsulinemia and require large doses of exogenous insulin. Our findings suggest that stabilization of claudin-19 may be a potential target for preventing DME.
